# Including the urban heat island in spatial heat health risk assessment strategies: a case study for Birmingham, UK

**DOI:** 10.1186/1476-072X-10-42

**Published:** 2011-06-17

**Authors:** Charlie J Tomlinson, Lee Chapman, John E Thornes, Christopher J Baker

**Affiliations:** 1 School of Civil Engineering, University of Birmingham, Edgbaston, Birmingham, B15 2TT, UK; 2 School of Geography, Earth and Environmental Sciences, University of Birmingham, Edgbaston, Birmingham, B15 2TT, UK

**Keywords:** Urban Heat Island, UHI, Birmingham, Experian, Heat Risk, Spatial Risk Assessment, GIS, Remote Sensing, MODIS

## Abstract

**Background:**

Heatwaves present a significant health risk and the hazard is likely to escalate with the increased future temperatures presently predicted by climate change models. The impact of heatwaves is often felt strongest in towns and cities where populations are concentrated and where the climate is often unintentionally modified to produce an urban heat island effect; where urban areas can be significantly warmer than surrounding rural areas. The purpose of this interdisciplinary study is to integrate remotely sensed urban heat island data alongside commercial social segmentation data via a spatial risk assessment methodology in order to highlight potential heat health risk areas and build the foundations for a climate change risk assessment. This paper uses the city of Birmingham, UK as a case study area.

**Results:**

When looking at vulnerable sections of the population, the analysis identifies a concentration of "very high" risk areas within the city centre, and a number of pockets of "high risk" areas scattered throughout the conurbation. Further analysis looks at household level data which yields a complicated picture with a considerable range of vulnerabilities at a neighbourhood scale.

**Conclusions:**

The results illustrate that a concentration of "very high" risk people live within the urban heat island, and this should be taken into account by urban planners and city centre environmental managers when considering climate change adaptation strategies or heatwave alert schemes. The methodology has been designed to be transparent and to make use of powerful and readily available datasets so that it can be easily replicated in other urban areas.

## Background

The aim of this paper is to integrate remotely sensed urban heat island data alongside commercial social segmentation data through a spatial risk assessment methodology in order to highlight potential heat health risk areas. This will build the foundations for a climate change risk assessment using the city of Birmingham, UK as a case study area.

### Heat Risk and Urban Areas

There is a growing recognition in the fields of bio-meteorology, epidemiology, climatology and environmental health that heat risk in urban areas is a problem, with literature considering cities in Europe [1], the USA [2,3], Australia [4] and Asia [5,6]. Elevated temperatures cause increased human mortality [7] which is exacerbated in heatwaves resulting in excess deaths. A number of examples are available in the literature such as in the 1995 UK heatwave [8], the 1995 Chicago heatwave [9] or the 2003 European heatwave [10] which affected France [11-14], England [15,16], the Netherlands [17], Portugal [18] and Spain [19]. There is growing evidence that the intensity, frequency and duration of heatwaves is likely to increase in the future [20]. This is prompting increased research into heat health risk projections [21,22], often as part of the broader remit concerning climate change and health [23-26].

The urban heat island (UHI) is a well documented phenomenon [27,28] that results in a conurbation being warmer than the surrounding rural areas. It is an example of an unintentional modification of the local climate and is principally caused by alterations to the energy balance influenced by variations of landuse, surface properties (e.g. surface roughness, albedo, emissivity) and geometry of the of the urban area [29,30]. Increased population in the city also promotes warming from anthropogenic heat release [31]. Hence, those that live in inner city areas are subsequently exposed to the UHI effect and can therefore be under increased heat health risk [2,8,32]. However, previous spatial risk assessment studies generally don't include the UHI [33]. With rates of urbanisation continuing to increase (the United Nations [34] predicting that population growth to 2050 will be absorbed exclusively in urban areas), the need for detailed heat risk assessments is paramount. Although this is an emerging research area [35,36], existing climate change work does not include a UHI component [37,38], despite it having a considerable influence on the mesoscale climate. Some work has been done to integrate the UHI within the United Kingdom Climate Projections 2009 (UKCP09) [39], but this is at a much larger scale than this paper considers. The result is a present need to integrate climate change projections with UHI data via a piecemeal methodology. Recent work utilising remote sensing techniques [40,41] has allowed the spatial extent of the UHI to be measured at a higher resolution than previously, and this paper focuses on using this data for heat health risk studies.

### Vulnerable Sections of the Population

There is evidence to suggest there are upper limits to human adaptation to temperature [42], which makes the consequences of increased temperatures important to understand. Although defining human thresholds for heat risk has many problems [43], it is possible to identify vulnerable groups (Table 1). High population density has been shown to correlate with areas of higher temperatures [44], and is to be expected given that high population density is often within inner city areas that are also impacted by the UHI. With specific reference to heat health risk, multiple studies have shown that increased population density results in increased risk [45-47]. Therefore it is reasonable to include people living in areas of high population density as vulnerable to heat risk.

**Table 1:** Groups vulnerable to heat risk

Vulnerable Group	References
Elderly People	[32,48-57]

Ill Health	[9,11,55,58,59]

High Population Density	[45-47]

High Rise Living	[9,32,59]

The elderly population has a relatively high percentage of illness and disability which increases their vulnerability [48]. Older, frail individuals are thought to have a lower tolerance to extremes of heat [49], and compounding factors, such as lack of mobility, further increase vulnerability [50]. This has been illustrated in the literature by studies in Switzerland [51], Italy [52], the Netherlands [53], Spain [54], Italy [55] and Latin America [56]. Within the UK, academic research [57] and the national Department of Health [32] recognise that the elderly are vulnerable to heat.

Another vulnerable group can be defined as those in "ill health". This includes those with pre-existing illness or impaired health, which could be physical or mental [58,59]. Known medical problems and those unable to care for themselves or with limited mobility are at increased risk [3,9,55], and diseases mentioned specifically include respiratory, cardiovascular and the nervous system [11].

People living on the top floor of flats or high rise buildings have also been found to have increased heat risk, with studies in Chicago in both 1995 [9] and 1999 [59] having similar results, finding that those living on higher floors were subject to increased risk. Within the UK, those in south facing top floor flats are classed as "high risk" by the Department of Health [32]. The reasons for this increased risk include the build up of temperatures in larger and taller buildings, and the increased exposure to incoming solar radiation resulting in higher temperatures.

Finally, young children are another group that could be at risk, with studies in Australia [60], America [61] and the UK [62] outlining the vulnerability of the very young. However, in this paper children have not been included because of the difficulties in locating detailed data (a consequence of the requirement to target parents or guardians in order to communicate). An effective way to reduce this research gap could be to target schools and embed heat risk education where appropriate.

### Spatial Risk Assessment Methodologies

The use of Geographical Information Systems (GIS) for spatial risk assessment work is a growing field, and covers a diverse range of hazards. These include various environmental hazards [63,64], flooding and geological hazards [65], technological hazard [66], hurricanes [67], fuel poverty [68] and many more. Work exploring spatial heat risk has so far been limited, but includes work in Australia [69], Canada [70] and the United States [71]. However the work that is most closely related to this paper is that of the field of climate change adaptation in the UK [33,72,73].

A critique of risk assessment methods in relation to climate change [74] details how problematic the process can be. However, given the increasing demand for "evidence based decisions" within governance, a form of risk assessment framework is required. The Adaptation Strategies for Climate Change in the Urban Environment (ASCCUE) project (more details available at http://www.sed.manchester.ac.uk/research/cure/research/asccue/) developed a risk assessment methodology based on "Crichton's Risk Triangle" [75]. This has been utilised in the UK as part of a broader methodology to assess flood hazard at both a neighbourhood and conurbation scale [65,73] and to assess heat risk in relation to climate change [33,72]. This paper builds on the methodologies developed in these papers and adds some important developments. In particular, this paper focuses on the impact of the UHI as well as developing objective methods that can easily be replicated nationally.

## Methods

### Study Area

The study area of Birmingham is the second most populous city in the United Kingdom, covering over 270 km^2 ^and with a population over one million [76]. Birmingham can be seen as representative of many inland mid-latitude cities worldwide, and using it as a case study offers a change from papers focussing on mega-cities such as London or New York which are too unique to have results which can easily be translated elsewhere.

This study utilises the "Lower layer Super Output Area" (LSOA) [77] as a spatial scale. LSOA is a geographical hierarchy designed for small area statistics, and although they do not have consistent physical size, they are not subject to boundary changes in the future, unlike other areas such as wards or postcodes. This makes them ideal for ongoing studies. A LSOA has a minimum population of 1,000 and an average population of 1,500, allowing data to be distributed easily without identifying individuals. As the LSOA is part of a hierarchy it is easy to change the scale, for example combining a number of LSOA into a single Medium layer Super Output Area (MSOA) which adds flexibility to the methodology as it allows comparison with datasets that may only be available at MSOA. There are 641 LSOA within the Birmingham area, numbered from 8881 to 9521 inclusive, with size (km^2^) ranging between 0.062 - 8.739, mean 0.418, standard deviation 0.541.

Health research with specific reference to the Birmingham area has taken place both within academia; exploring the relationship between mortality and temperature [78], looking at the 1976 heatwave [79] and through the public sector; looking at climate change and health [80]. This previous work has not included a spatial aspect, which is an important research gap given the size and diversity within Birmingham, and particularly when including a UHI component. Detailed work on Birmingham's UHI has recently been undertaken [41] and data is readily available, allowing this important effect to be considered in detail.

### Spatial Risk Assessment

The methodology utilised in this paper has deliberately been kept simple and transparent in order to remove excessive complicated jargon and help explanation to stakeholders including local authorities. However, at this stage it is important to clarify the terminology used in this paper, as throughout the risk assessment literature there are various terms that have multiple definitions.

**Figure 1:**
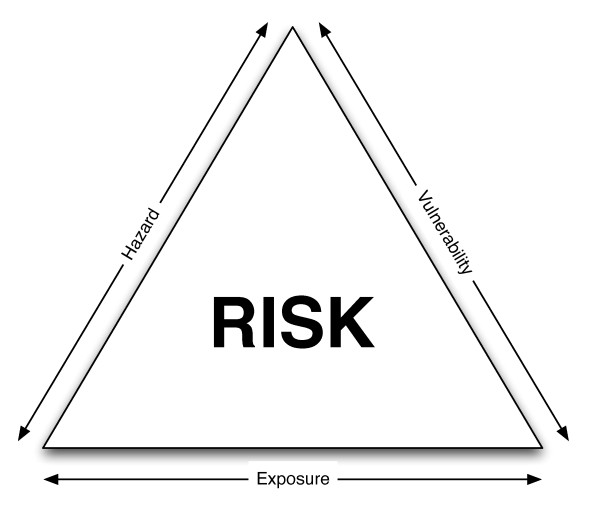
**Crichton's risk triangle (from **[73] and [75]).

**Figure 2:**
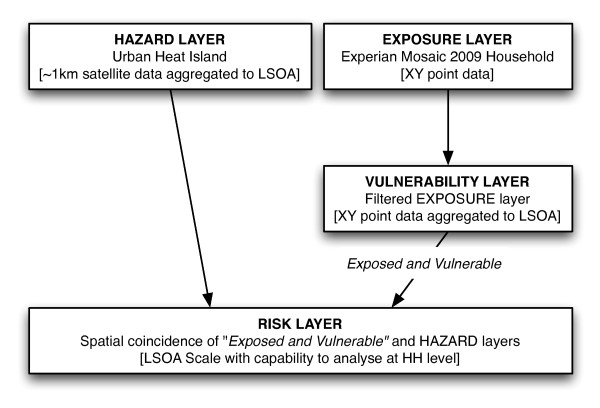
**Simplified flowchart of GIS spatial risk assessment methodology (adapted and developed from **[73]).

The main risk assessment theory focuses on "Crichton's Risk Triangle" (Figure 1) that states that risk is a function of hazard, exposure and vulnerability, and all must be spatially coincident for a risk to exist. The advantages of splitting the definition are that it makes the process clear and transparent and simplifies analysis within a layering system in a GIS. A hazard is something that may cause a risk, and in this method the spatial and temporal aspects of the hazard are required, alongside the magnitude. This could be historical, measured or predicted, and in this case the increase in temperature from the UHI is being considered, measured from remotely sensed satellite data. The exposure represents what is exposed to the hazard and at a basic level is simply a spatial coincidence between the hazard and the exposure of interest. Various items could be exposed and relevant data about each is required spatially for this method to be useful. Examples could be buildings (with corresponding metadata such as types or value) or people (with metadata such as age or health problems) and this paper uses high resolution commercial social segmentation data. Vulnerability refers to which aspects of the exposed elements are vulnerable to a given hazard, and this is generally defined by referencing a vulnerability table. Certain groups are more vulnerable to heat risk, for example the elderly. The final risk layer is generated from the spatial coincidence of the hazard layer and the *exposed and vulnerable *layer. This is a simplification of the ASCCUE work and a flowchart visually illustrates the workflow (Figure 2). These methodological changes, which remove the "hazard-exposure" layer and place more emphasis on the "*exposed and vulnerable"*, were chosen due to simplification of data manipulation and ease of explaining to stakeholders. A more detailed explanation of Crichton's risk triangle and real world examples of use are available [33,65,72,73,75]. In order to spatially represent each of the hazard, exposure, vulnerability and risk layers a coherent spatial scale is required across all layers. All items of interest are merged at the LSOA scale.

A standardisation technique has been employed, in order to illustrate each variable on the same scale and ensure ease of combining layers of a different nature. This technique helps quantify the process and enables statistical analysis and comparisons to be carried out more effectively. This is based on the Hazard Density Index (HDI) [66] that a number of studies have used successfully [63,81]. Individual variables are standardised by dividing each variable value from the maximum value of that variable across the complete study area. The formula used is: "*LSOA score/max LSOA score across Birmingham = standardised score for each LSOA*". This standardises the variable to between zero (low) and one (high).

When combining layers it is possible to vary the weighting of values based on relative importance. However, in this paper all weightings have been kept equal in the interests of transparency. Other studies have used equal weighting methods with success [63,64]. If weighting of values is varied the process becomes subjective and the resultant maps open to manipulation. Appropriate use of weightings requires considerable knowledge concerning all the variables and techniques. It is anticipated that the results of this work will be incorporated into a spatial decision support tool where the weightings can be altered according to specific user requirements.

### Hazard Layer: Urban Heat Island

High resolution UHI mapping can be obtained through remote sensing methods, including airborne (such as NASA's ATLAS sensor [82]) or satellite platforms. The highest resolution (~60 m) satellite sensors used for UHI work include Landsat ETM+ [83] and ASTER [84]. This paper uses the Moderate Resolution Imaging Spectroradiometer (MODIS) on NASA satellites (due to the increased temporal coverage and thermal accuracy) to measure Land Surface Temperature (LST) at a ~1 km resolution on cloud free days and this has been analysed and manipulated (full details available [41]) in order to measure the magnitude of the surface UHI. The relationship between LST (and therefore surface UHI) and measured air temperature is complicated, with techniques such as statistical regression [85], solar zenith angle models [86] or thermodynamics [87] often used to explore the relationship. LST and air temperature are not directly comparable, however in the case of the UHI, it is reasonable to believe that spatial trends will be similar when comparing LST and air temperature, and therefore remotely sensed data is a useful dataset as absolute values are not vital in this methodology.

Detailed UHI work has been carried out for Birmingham [41] and it is this dataset that has been used in this paper. The MODIS remotely sensed image of the night of the 18^th ^July 2006, used as a "heatwave" example was resampled and then zonal statistics were carried out in order to facilitate generalisation at the LSOA scale. The mean UHI magnitude (°C) for each LSOA was taken to standardise the LSOA output on a scale between zero and one, as for other layers. The resultant layer illustrates the spatial pattern of the UHI across the conurbation on a specific heatwave day, representative of a day with ideal conditions for UHI generation (low windspeed and low cloud cover). However the spatial pattern of the UHI has been shown to be similar across a number of different meteorological conditions [41].

The main alternatives to satellite data for calculating the UHI include ground sensor measurements or model output. There is a paucity of ground sensors in Birmingham, and other approaches (for example transect based [88]) require extensive fieldwork. UHI model's [36,89] have been developed, but require considerable work to collate accurate input variables and validate the results. Satellite data is readily available globally, increasing the utility of the methodology.

Overall, the inclusion of the UHI as the hazard layer explicitly fills a specific research gap from other heat risk studies. The work could be expanded on, for example to include the possible effects of both climate change and the UHI, however this is outside the scope of this paper.

### Exposure Layer: Experian Mosaic 2009 Data

The exposure layer in this paper is made up of detailed commercial data from Experian on every household in Birmingham. Experian are a global company focussed on providing information to help business and in the UK they are commonly known for being one of the three credit reference agencies the financial industry uses. Within this paper, the Experian Mosaic UK 2009 product is used which is a consumer classification for the United Kingdom, providing "an accurate understanding of the demographics, lifestyles and behaviour of all individuals and households in the UK" [90], classifying each household into one of 15 groups, and below that one of 67 types. This exact method is suitable for the UK, but Experian have a number of consumer segmentation products for 29 countries that classify over a billion consumers, so it could be easily adapted to other parts of the developed world. The Mosaic classification is built using 440 data elements, and is updated and verified bi-annually [90].

The Mosaic 2009 dataset was supplied for all of Birmingham at household (HH) level, with each HH including attributes of X and Y location, Mosaic Type and Mosaic Group. For the purposes of this paper, HH data is generally aggregated up to LSOA levels as this can be distributed without personal identities being disclosed, whilst still giving a relatively high resolution. However, having access to the HH data gives additional flexibility both for the methodology and analysis. Supplied alongside the raw data was the key to Mosaic types, a document that gave in depth qualitative information for each Mosaic type, including a general overview followed by specific demographic information related to where the type lives, how they live, world views, financial situation and online behaviour. Using a single dataset to underpin the methodology and analysis was a deliberate choice, designed to remove problems of availability and contextual differences that have been illustrated in previous studies [63]. The data used in this project is at HH level, and details the 427,914 HH contained within Birmingham city extents. Experian offer a risk dataset (Perils), encompassing flood, subsidence, windstorm and freeze risk [91] however heat risk is notably absent, and therefore this work also acts as a proof of concept for expanding Experian's risk dataset product portfolio. The exposure layer is point shapefile with one point for each household containing attribute data including Mosaic type; data is summarised into LSOA at a later stage using GIS techniques. Titles of the Mosaic types used in this paper are detailed in Table 2, and more details are available in the Mosaic 2009 brochure (available online [90]).

**Table 2:** Titles of relevant Mosaic type identified for specific vulnerabilities

Mosaic Number	Mosaic Titles	Vulnerability
20	Golden Retirement	Elderly

21	Bungalow Quietude	Elderly

22	Beachcombers	Elderly

23	Balcony Downsizers	Elderly

38	Settled Ex-Tenants	Ill

39	Choice Right to Buy	Ill

42	Worn-Out Workers	Ill

43	Streetwise Kids	Ill

44	New Parents in Need	Ill

45	Small Block Singles	Ill

47	Deprived View	Ill

50	Pensioners in Blocks	Elderly

51	Sheltered Seniors	Elderly

52	Meals on Wheels	Elderly

53	Low Spending Elders	Elderly

65	Anti-Materialists	Ill

*All 67 Mosaic types were used to calculate density and high rise vulnerabilities

An alternative data source is the British Census (a decadal survey of every person and household in the UK), and this has been used in other studies [33,57]. However, it will take time for data from the recent 2011 Census to become available after being verified and quality assured, and available data from the 2001 Census is now outdated. This paper does not use Census data, given the time delay and the future uncertainty over the survey given the current governmental spending cuts. Mosaic uses current year estimates of Census data for 38% of the information used to create the classification, alongside additional datasets and verification. This makes the data more useful as it is upto date. For more information on the classification system, see the brochure online [90].

### Vulnerability Layer(s): Specific Vulnerable Types

The vulnerability layer in this paper is made up of vulnerable types extracted from the exposure layer, made up of Experian Mosaic HH types. Vulnerable types have been defined through a literature review and justifications for each layer are given in Table 1. The following details how each specific vulnerable type was identified and extracted from the data available in the Mosaic dataset.

Elderly people were identified as Mosaic group E, "Active Retirement" (type 20,21,22,23) and L, "Elderly Needs" (type 50,51,52,53). Within these groups, there is a wide range of socioeconomic factors, however all are elderly. The literature identified elderly as a vulnerable type, and whilst affluence can reduce vulnerability, for example by financing air conditioning units, it cannot totally mitigate the vulnerability. The number of HH classed as "elderly" per LSOA was counted and then standardised as discussed.

Other heat risk studies [33] discuss how analysing flats or high rise buildings could be a possible addition to their study. This paper uses a combination of datasets to calculate people living in high rise buildings. The Mosaic data gave household locations (including multiple households at the same XY coordinates). Ordnance Survey Mastermap, the highest resolution vector mapping solution available in the UK, details individual buildings at polygon level. Individual building polygons across Birmingham were extracted from Mastermap, and then the number of HH points falling within each polygon was counted. This was then filtered to show only polygons with greater than ten HH within. The rationale behind this number is that buildings with less than ten households are not likely to be sufficiently high rise. This number would be easily altered for use in different cities. Light Detection and Ranging (LIDAR) height data could be combined in order to obtain true height of buildings but this approach was not used because this methodology focuses on using Experian data for ease of repeatability.

Density of households per LSOA was calculated simply by using following formula, for each LSOA "*HH density per LSOA = number of HH in LSOA/area of LSOA (km*^*2*^*)*". The result is household density per km^2 ^that was standardised as per the technique already detailed.

The vulnerable group "ill health" was created by a literature and keyword search of the Mosaic 2009 key document for keywords "health" or "illness" followed by qualitative interpretation of the results by a single interpreter to avoid bias. This identified Mosaic types 38, 39, 42, 43, 44, 45, 47, and 65 as including people with ill health. Not all HH will be of ill health, but examples of the way these groups are described includes "they have health problems " or "higher levels of illness" or "many have health issues, including mental health issues". The number of HH classed as "ill health" per LSOA was counted, and then standardised as described.

### Risk Layer

**Figure 3:**
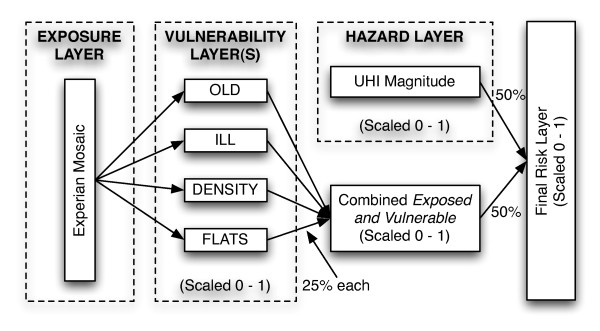
**Detailed flowchart of spatial risk assessment methodology**. All are at LSOA level except the Exposure Layer which is HH points.

To create the final risk layer, the four vulnerability layers were combined into a single "*exposed and vulnerable" *layer (each weighted at 25%) which was then spatially combined with the hazard layer (each weighted at 50%), a technique that has been used successfully for previous spatial risk assessment [63]. This process is illustrated in Figure 3.

## Results and Discussion

When interpreting the results it is important to note that when generalising at the LSOA scale, some data will be masked in a small number of cases. For example, the Sutton Park area in the north of the city that contains the actual park has to be extended to include an area with approximately 1,500 people in order to match the LSOA geography. As this LSOA is physically one of the biggest by area within Birmingham, maps can look skewed.

### Spatial Trend between the UHI and Exposed and Vulnerable

**Figure 4:**
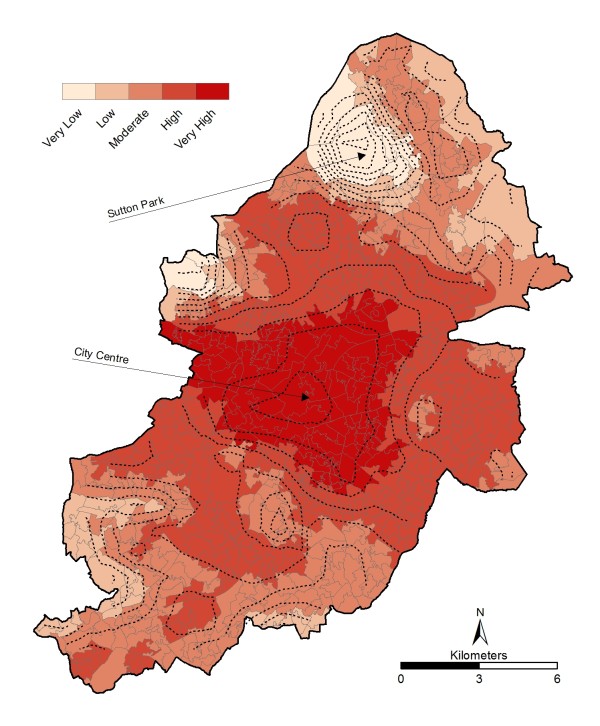
**Birmingham UHI under heatwave conditions at LSOA level**. 18^th ^July 2006 from MODIS remotely sensed data. Shown with contour lines for validation.

The UHI under heatwave conditions at LSOA level (Figure 4) reflects the results (from [41]) and gives confidence that the generalisation to LSOA has not compromised the dataset. A full discussion of the spatial trends is available [41] but in summary, the highest temperatures are found in the city centre where as the Sutton Park area in the north of the city is the coolest area. As expected, there is a general trend towards lower temperatures in the suburban areas.

**Figure 5:**
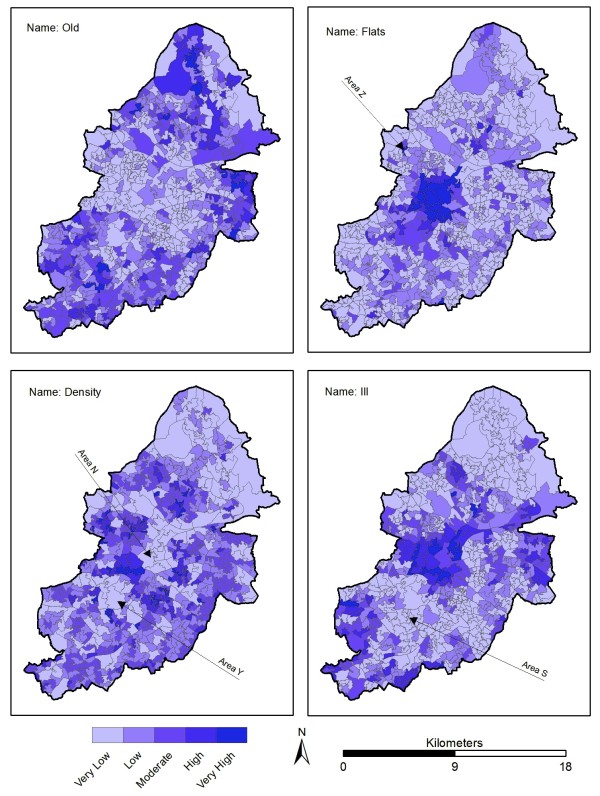
**Four "*exposed and vulnerable*" layers at LSOA level**. Named areas are detailed in text.

The four main "*exposed and vulnerable" *layers were displayed in a GIS with natural breaks (Jenks) symbology (Figure 5) in order to view groupings inherent in the data. Concentrations of old people are scattered throughout the city, with distinct clusters in the north. This is not surprising as the northern Sutton Coldfield area is generally regarded as having a slower pace of life, with close proximity to countryside being appealing to the older generation. This also helps explain the lack of elderly people in the city centre, where they are conspicuously absent. There are additional concentrations of older people in the east and towards the south.

Conversely when looking at flats, there is a significant concentration in the city centre, a result of high land costs forcing the development of high rise flats. This property type is unappealing for the majority of elderly people, given the difficulties of access (e.g. stairs/lifts) and greater noise levels. Away from the centre, there are other LSOA's with high levels of flats, including small numbers in the north, and even less in the south. For example, clusters can be found in student areas, such as the high rise student housing located on Birmingham City University campus (Area Z, Figure 5).

There is less of a visible range when looking at density (detailed in HH per km^2^). Again, the highest density LSOA's are located in the city centre, extending north westwards into areas renowned for having a high immigrant population. Conversely, density reduces heading south from the city. For example, Edgbaston (Area Y, Figure 5) is an affluent area that also includes the University of Birmingham, Edgbaston golf course and other land uses not associated with households. The north east quarter of the city centre (Area N, Figure 5) is also low density, and is an area traditionally associated with industry. However, the overall density levels across the city are generally similar, with local variations between LSOA's dependent on the presence of greenspace (which increase the size of the LSOA area but not numbers of HH).

Finally, significant concentrations in the spatial pattern of people with ill health exist. This is particularly evident across the city centre and in a belt north east of the city centre and towards the cities eastern edge. Pockets are also visible in the south, after noticeable lows in the affluent area of Edgbaston and the transient student population of Selly Oak (Area S, Figure 5), who are unlikely to stay in the same place long enough for reliable health statistics to be compiled.

A Spearman's rank order correlation was carried out to determine the statistical relationships between each "*exposed and vulnerable*" group and the UHI at the LSOA level (*n = 641*). Table 3 shows that the results generally agree with the visual interpretation and all relationships are statistically significant (*p < 0.01*) except density *vs *flats. There is a weak positive correlation between density, flats and illness with the UHI, showing that as the UHI increases, the number of "*exposed and vulnerable*" groups also increases. There is a stronger negative correlation between old people and the UHI that agrees with the visual interpretation already discussed.

**Table 3:** Spearman's rank correlation coefficient matrix

	Density	Flats	Ill	Old
Density	-	-	-	-

Flats	0.058*	-	-	-

Ill	0.161**	0.254**	-	-

Old	-0.256**	0.241**	0.158**	-

**UHI (mean)**	0.329**	0.125**	0.224**	-0.396**

* Correlation is not significant at the 0.01 level

** Correlation is significant at the 0.01 level

**Figure 6:**
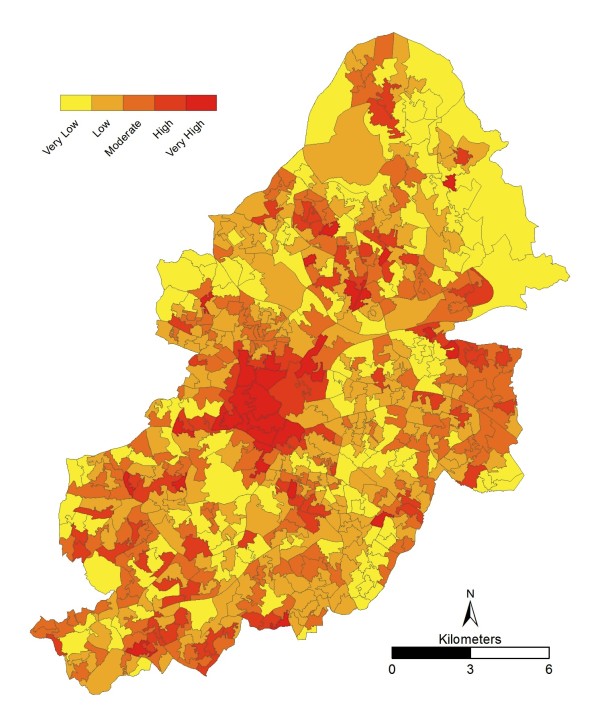
**Combined (equal weighting) "*exposed and vulnerable*" layer at LSOA level**.

When the above four vulnerable groups are combined and equally weighted (Figure 6) it is clear to see that the very high risk areas are concentrated around the city centre. This is to be expected due to the individual distributions already discussed, and agrees with previous work in the USA which has found that vulnerability increased in warmer neighbourhoods [45] and that these neighbourhoods had a tendency to be located within the inner city [71]. Although equal weightings for all layers have been used in this study, it is recognised that features of urban form (e.g. density) can also act as predictors for the UHI. As a result, this can impact the output risk, and is an area that could be explored more in the future when considering different weightings for layers.

### The Final Risk Layer

**Figure 7:**
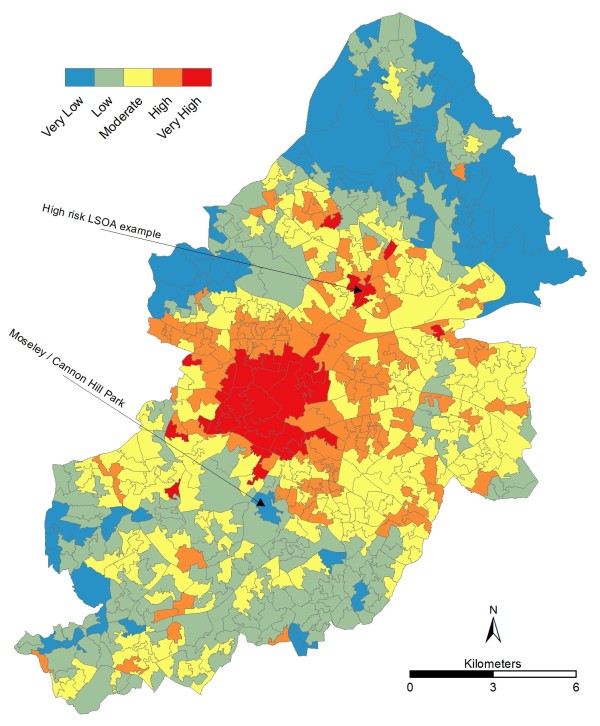
**Final risk layer at LSOA level**.

Figure 7 shows that the majority of the "very high" risk LSOA's are grouped together in the city centre. It is here where the highest temperatures are experienced as well as the highest number of ill people, number of flats and density. However, additional pockets of "very high" risk also exist and these require additional explanation. As already discussed, a high concentration of flats increases the density of a LSOA. Outside of the city centre, these flats are frequently high rise social housing that is often associated with increased illness in the poorer sections of communities. A typical "high risk" pocket has significant high rise social housing which increases the density, scores highly for flat and often for illness as well.

The lowest risk areas are found in the north west (Sutton Park area) and north east of the city. This is explained by the low and very low UHI risk coupled with very low "*exposed and vulnerable*" populations. An anomaly of this area is that it actually has the highest concentration of elderly people, but they are less vulnerable to heat due to their distance from the city centre. Other very low risk areas are evident west of the city centre and scattered south of the city centre. In general these are heavily linked to greenspace; which has the dual effect of ameliorating the UHI and reducing the number of people living in an area. Indeed, a more explicit look at the distribution of greenspace within the conurbation could be useful (e.g. using surface cover analysis [92] or energy exchange models [93]), given the benefits of reducing the UHI [94] and improving health inequalities [95].

### Household Level

**Figure 8:**
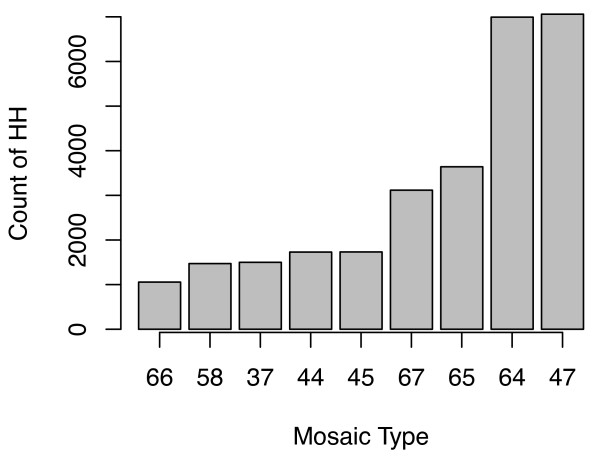
**Mosaic type within "very high" risk LSOA**. Filterered to only include HH counts > 1000.

**Figure 9:**
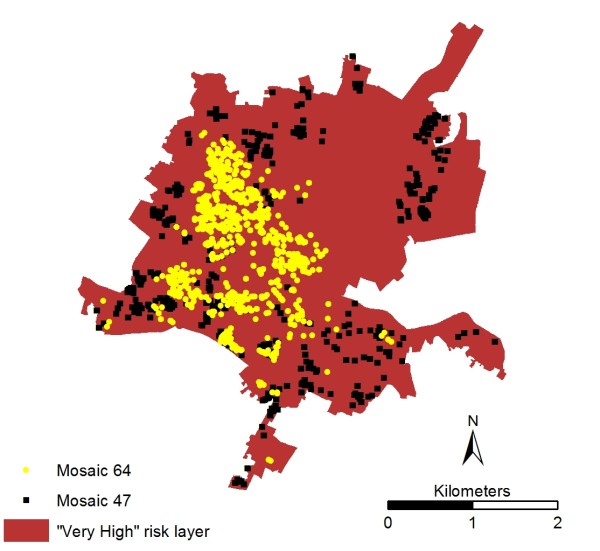
**Mosaic type 64 and 47 spatial distribution within "very high" risk city centre area**.

A strength of the methodology detailed in this paper is that once the risk areas have been identified, a subsequent detailed analysis down to HH level can be conducted. Such high resolution work within urban areas is a logical development of previous broader scale work, such as the province wide analysis carried out in Quebec, Canada [70]. A GIS was used to identify 37,477 HH's (or ~8.76% of 427,914) that fall within the "very high" risk LSOA's (33 out of 641). These HH's can then be profiled using Mosaic type (Figure 8), which illustrates the vast majority are either 47 (Deprived view) or 64 (Bright young things), accounting for ~7,000 HH each. This illustrates a clear divide within the "very high" risk area which is only able to be explored by having access to high resolution underlying datasets such as Mosaic. Type 47 are "poor people who live in high rise blocks of socially owned housing...many have disabilities...characterised by extreme poverty". Type 64 are "well educated young high flyers...live in smart inner city areas...mostly modern, purpose built or converted apartments". Despite living in broadly the same area, the populations are generally separated (Figure 9) and are at polarised levels of heat risk. Type 64 typically live in new apartments located within the inner city. These dwellings may have good insulation, air conditioning or even passive cooling. This is a contrast to type 47, who live in older, social apartments located in less desirable areas surrounding the urban core. Unlike type 64, this group is unlikely to have the finances available to make themselves comfortable or safe.

## Conclusions

This study has illustrated a simple methodology for quantifying risk, through a process where each stage can clearly be explained and understood. It offers suggestions for the output to be customised, for example with different weightings or replacement with different hazards or risk groups as appropriate. This work offers the foundations for a spatial decision support tool that could be linked to climate change and projection models in order to consider climate change adaptation with a focus on heat health risks. Indeed, such data is potentially of great use to local authorities and health agencies when deciding on targeted campaigns.

The highest vulnerability is shown to exist in the inner city areas. This result agrees with similar work done in the USA [45,71] and is a direct consequence of the increased temperatures associated with the UHI in this area. Furthermore, many of the root causes of the UHI (for example lack of greenspace, high anthropogenic heat output, significant built form) can be linked to vulnerable groups and therefore a feedback loop is created.

The simplicity of the methodology could be significantly refined through further research. For example, throughout this paper no explicit temperature values have been mentioned. This is deliberate as the focus has been the spatial identification of risk groups. This paper assumes that a single day "snapshot" of UHI data is representative of varying conditions, but an alternative heat hazard layer could be developed using outputs from UHI models, which would allow for flexibility when considering varying conditions.

A significant research gap in this paper is the verification of the results, for example against health and mortality records in association with previous heat events (e.g. heatwave events in 2003 or 2006). This is the focus of ongoing work, but the data is presently not available at both a high temporal and spatial scale, which would be required in order to test for links at LSOA level. The data that is available is of limited utility as it is hard to quantify heat related health issues or mortality with any degree of certainty, and records have unreliable spatial attributes; in that they may relate to a patients home or to the hospital, and significant distances may be present between these. Hospital discharge data could potentially help quantify heat-related health admissions, although again the utility may be restricted due to small datasets and restricted availability.

In summary, the methods shown offer a repeatable methodology that can be utilised in many countries. This is made possible by the flexibility of a GIS based approach, the worldwide availability of the MODIS satellite data and the significant coverage of Experian's segmentation data throughout the developed world.

## List of Abbreviations

ASCCUE: Adaptation Strategies for Climate Change in the Urban Environment; GIS: Geographical Information System; HDI: Hazard Density Index; HH: Household; LSOA: Lower layer Super Output Area; MODIS: MODerate resolution Imaging Spectroradiometer; MSOA: Middle layer Super Output Area; UHI: Urban Heat Island; UKCP09: United Kingdom Climate Projections 2009; UMT: Urban Morphology Type.

## Competing interests

The authors declare that they have no competing interests.

## Authors' contributions

CJT carried out the risk analysis and drafted the manuscript. LC, JET and CJB offered advice throughout the research and feedback on the draft manuscript. All authors read and approved the final manuscript.
